# Analysis of Clinical Characteristics of Immune-Related Dry Eye

**DOI:** 10.1155/2017/8532397

**Published:** 2017-05-30

**Authors:** Hua Wang, Ping-Bao Wang, Ting Chen, Jing Zou, Ya-Jia Li, Xu-Fang Ran, Qiang-Xiang Li

**Affiliations:** ^1^Ophthalmology Department, Xiangya Hospital, Central South University, Changsha, Hunan Province 410008, China; ^2^Hunan Provincial People's Hospital, Changsha, Hunan Province 410016, China

## Abstract

**Aim:**

To discuss the clinical characteristics of immune-related dry eye.

**Methods:**

Simple dry eye (SDE) group: we selected 224 patients of simple dry eye with no systemic lesions. Immune-related dry eye (IRDE) group: we selected 207 patients of dry eye complicated with immune system diseases, including 70 cases of Sjögren's syndrome (SS), 72 cases of systemic lupus erythematosus (SLE), and 65 cases of rheumatoid arthritis (RA). The classification of all patients was performed. The difference between the two groups was compared, including age, gender, ocular surface fluorescein staining and inflammatory reaction, tear breakup time (TBUT), Shirmer I test, confocal microscopy scan, and dry eye grading.

**Results:**

Compared with the SDE group, the patients of IRDE group were younger (*P* < 0.05). The female patients were significantly more than the male ones (*P* < 0.05). Corneal staining counts and ocular surface inflammation were significantly increased (*P* < 0.05). TBUT and Shirmer I test shortened significantly (*P* < 0.05). Corneal nerve fibers were less, and the number of local lymphocyte was significant increased. The number of dry eye patients in the moderate or above IRDE group was significantly increased (*P* < 0.05).

**Conclusions:**

The dry eye symptom and sign and ocular surface inflammation of IRDE were significantly more severe than those of the SDE.

## 1. Introduction

Dry eye is a multifactorial disease in which tears and ocular surface are accompanied with the characteristic symptoms of discomfort, visual disturbance, and tear film instability as well as increased permeability of tear film and ocular surface inflammation [1–3]. In recent years, with the aging of the population and the wide spread use of electronic terminal products, the dry eye prevalence rate increased significantly and the onset of the disease also shows a trend of getting younger. Among the patients with dry eye syndrome, some patients are complicated with the systemic immune system diseases. The dry eye symptoms and signs of such patients are often more serious, the treatment effect is poorer, and the prognosis is not ideal. There are clinical characteristics of immune-related dry eye. The aim of this study was to analyze and discuss the clinical characteristics of dry eye caused by immune-related diseases. The prevalence rates of primary Sjögren's syndrome (SS), systemic lupus erythematosus (SLE), and rheumatoid arthritis (RA) are the highest in systemic immune diseases. The patients with SS, SLE, and RA simultaneously complicated with dry eye diagnosed by the rheumatic immune outpatient in our hospital were selected to compare with the patients with simple dry eyes not complicated with the systemic immune diseases and other local lesions of eyes. The clinical characteristics of dry eye in patients with autoimmune diseases were discussed in this paper.

## 2. Subjects and Methods

### 2.1. Selection of Subjects

This is a prospective study. We have obtained prior approval from the ethics committee before the study. The dry eye patients were diagnosed in the rheumatic immunology and ocular surface outpatients of our hospital from January 2012 to December 2014 were selected. They were divided into two groups in this study: (a) simple dry eye (SDE) group consisted of 224 patients with simple dry eye no systemic association, primary Sjögren's syndrome, blepharitis, and Meibomian gland dysfunction (MGD), including 86 male patients and 138 female patients, aged 18–78 years, with an average age of 53.6 ± 20.6 years and (b) immune-related dry eye (IRDE) group consisted of 207 patients with dry eye complicated with the rheumatic immune diseases such as SS, SLE, and RA, including 70 cases of SS, 72 cases of SLE, and 65 cases of RA. Among them, there are 22 male patients and 185 female patients, aged 22–57 years, with an average age of 42.8 ± 18.3 years.

#### 2.1.1. Inclusion Criteria

The inclusion criteria are the following: (1) according to the dry eye diagnosis criteria developed by the International Dry Eye Work Shop (DEWS) in 2007, it can be diagnosed as dry eye both SDE group and IRDE group if it has subjective symptom of dry eye and meets any of the following circumstances: (a) tear breakup time (TBUT) ≤ 10 sec, (b) Schirmer I test ≤10 mm/5 min, and (c) conjunctival or corneal fluorescein staining positive. (2) Dry eye patients diagnosed by the rheumatic immunology as either of SS, SLE, and RA are included into IRDE group. (3) The dry eye patients not complicated with systemic immune disease and local ocular diseases are included into the SDE group.

#### 2.1.2. Exclusion Criteria

The exclusion criteria are the following: (1) history of ocular surgery, ocular trauma, and systemic and local ocular medications; dry eye treatment including using artificial tear; and immunosuppressive treatment including using steroids, cyclosporine, and FK506; (2) other ocular lesions, such as MGD, blepharitis, glaucoma, lid abnormalities, conjunctival chalazion, and contact lens users; (3) complicated with other systemic lesions, such as diabetes and hyperthyroidism; and (4) psychological or psychiatric diseases.

### 2.2. Comparative Analysis of Symptoms and Signs

The dry eye classification [1, 2] of all patients was performed in line with the dry eye diagnosis and treatment guideline developed by the DEWS. The difference was compared between two groups, including age, gender, conjunctival and corneal fluorescein staining, TBUT, Schirmer I test, conjunctival congestion, papillary and follicular hyperplasia, secretions, corneal nerve fibers and the number of local lymphocyte, and dry eye severity (dry eye grading). According to the classification of dry eye severity performed by DEWS in 2007, dry eye is classified into four levels [1], namely minor, moderate, severe, and extremely severe [1]. The dry eye reference diagnosis criteria proposed by Liu [3] was used as the scoring criteria of conjunctival and corneal fluorescein staining. The cornea was divided into four quadrants by the assessment method. The fluorescent staining of each quadrant is classified into four grades, that is, null, minor, moderate, and severe, and 0–3 points, respectively, and then the fluorescent staining of the whole cornea is scored as 0–12 points. The order of the examination is TBUT by using fluorescein paper firstly, then conjunctival and corneal fluorescein staining and Schirmer I test by using Schirmer filter paper lastly. In the outcome of TBUT, conjunctival and corneal fluorescein staining and conjunctival congestion, papillary and follicular hyperplasia, secretions were observed by using slim lamp microscope. Corneal nerve fibers and the number of local lymphocyte by confocal microscopy scan.

### 2.3. Statistics Methods

SPSS17.0 software was used for the descriptive statistical analysis of the experiment results. Through the normality test, the sign data of the two groups tend to a skewed distribution, so the median (*M*) ± interquartile range (*Q)* describe the centralized location and discrete degree of the statistical data. The *t*-test was used for the comparison of dose data between two groups, and chi-square test was used for the comparison of the count data and total rate or composition rate between two groups. It is considered that there is a statistical difference as *P* < 0.05.

## 3. Results

### 3.1. Comparison of General Data

Comparison of gender and age: the incidence rate of female patients was significantly higher than that of the male patients (*P* < 0.05) among the dry eye patients of the two groups; such a difference was more significant in IRDE group (*P* < 0.005). Compared with the SDE group, the patients of the IRDE group were younger (*P* < 0.05) (see Table 1).

### 3.2. Comparison of Signs

**Figure 1:**
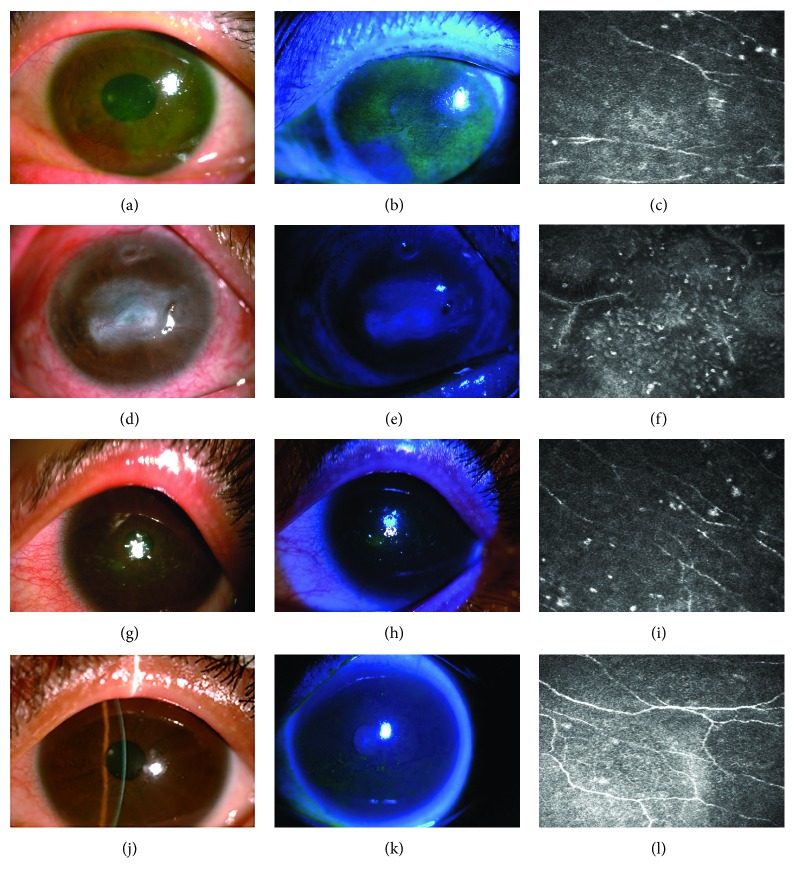
Ocular surface photograph, fluorescein staining, and confocal microscopy scan. (a1), (a2), and (a3) are the case of SS. (b1), (b2), and (b3) are the case of RA. (c1), (c2), and (c3) are the case of SLE. (d1), (d2), and (d3) are the case of SDE.

TBUT of the two groups was significantly shortened, but there was no difference between the two groups (*P* > 0.05). Schirmer I test of the two groups was significantly shortened, and the shortening of the IRDE group was more significant than that of the SDE group (*P* < 0.05). There was an abnormal positive staining for the corneal fluorescein staining of the two groups, and the corneal staining score of the IRDE group was higher and there was a significant difference between the two groups (*P* < 0.05). Corneal nerve fibers were less, and the number of local lymphocyte was significantly increased in IRDE by confocal microscopy scan (see Table 2 and Figure 1).

### 3.3. Comparison of Ocular Surface Inflammatory Reaction

The incidence rate of conjunctival congestion, conjunctival papillary, and follicular hyperplasia of IRDE group was significantly higher than that of the SDE group (*P* < 0.05). There was no significant difference of incidence rate of conjunctival sac secretions between the two groups (*P* > 0.05). See Table 3.

### 3.4. Comparison of Dry Eye Classification

In SDE group, most of patients belonged to minor or moderate dry eye, accounting for 73.2%. In the IRDE group, most of patients belonged to the moderate or above dry eye, especially the incidence rate of severe and extremely severe dry eye was significantly higher than that of the SDE group (*P* < 0.05). In the IRDE group, the incidence rate of severe and extremely severe dry eye for SS was significantly higher than that for SLE and RA (*P* < 0.05) (see Table 4).

## 4. Discussion

The SS, SLE, and RA are all the autoimmune diseases with a higher incidence rate and often involve in the connective tissues of the skin, mucous membrane, glands, and so forth [4–8]. Hence, the cornea, conjunctiva, and lacrimal gland tissues of the ocular surface have also become the immune attack positions, which lead to the damage of their structures and functions and cause the severe ocular surface problem, for example, dry eye. Compared with the simple dry eye, the immune-related dry eye has also its special clinical manifestations in addition to the common features of the dry eye diseases.

It was found in this study that the incidence rate of simple dry eye and immune-related dry eye in women was significantly higher than that in men, and this difference was more significant in the immune-related dry eye, which may be associated with such a fact that the incidence rate of dry eyes and immune diseases in women is significantly higher than that in men [9]. Most of the patients with simple dry eye were middle-aged and elderly women. However, with the popularity of video terminal equipment such as computers and smart phones, the incidence rate of simple dry eye in men and youth crowds also appeared to rise year by year [10–12]. Most of the patients with immune-related dry eye were middle-aged people and the youth, which are much younger than those with simple dry eye.

In the aspect of clinical signs, TBUT and Schirmer I test of the two groups of patients were all shortened, but the TBUT difference of two groups was not significant. On the contrary, the shortening of Schirmer I test in the immune system-related dry eye group was more significant and there was a statistical difference. This result implied the dry eye with insufficient tear secretion because of the autoimmune diseases involved in the ocular surface gland tissues such as lacrimal gland and accessory lacrimal gland, leading to the significant reduction or insufficiency of tear secretions [13–16]. The positive rate of corneal fluorescein staining in immune-related dry eye was higher and more serious. It showed that the autoimmune diseases were more likely to cause the epithelial damage of cornea, conjunctiva, and other connective tissues, and damage of this kind was more serious [17, 18].

The comparison of ocular surface inflammatory reactions of the two groups also showed that the incidence rate of conjunctival congestion, conjunctival papillary, and follicular hyperplasia of the immune-related dry eye was significantly higher and conjunctival sac secretions was lesser, belonging to noninfectious inflammation. This showed that the ocular surface inflammatory reaction of this kind of patients was more serious than that of simple dry eye [19–23]. In this study, we also found that corneal nerve fibers were less and the number of local lymphocytes was significantly increased in IRDE than those in SDE by confocal microscopy scan. This showed that immune reaction of autoimmune disease can promote and increase local lymphocyte inflammatory infiltration and reaction of ocular surface and would seriously damage the corneal nerve fibers.

The comparison of severity classification of dry eye of the two groups showed that most of patients with simple dry eyes belonged to minor-moderate dry eyes; the immune-related dry eyes often resulted in the moderate dry eye or above, especially the incidence rate of severe and extremely severe dry eyes significantly increased. In the three major immune system diseases of SS, SLE, and RA, the incidence rate of moderate and severe dry eyes in SS patients was higher and its clinical manifestation was more serious.

Based on the results of this study, IRDE were presented more in female patients than in male patients. The dry eye symptom and sign and ocular surface inflammation of IRDE was significantly more severe than that of the SDE. We can reasonably infer that, on one hand, the autoimmune disease can attack the lacrimal gland tissue via immune reaction to achieve the reduction of the tear secretion; on the other hand, the autoimmune disease can attack the ocular surface epithelial cells and promote the ocular surface inflammatory reaction to cause the damage of accessory lacrimal glands and goblet cells and promote the occurrence and growth of ocular surface inflammatory reactions and result in the damage of ocular surface epithelial cells and the reduction of mucin secretion. The integrated action of multiple factors and pathways enables the immune-related dry eye to become a serious mixed type of dry eye [24–29].

The research results showed us clinically that it was necessary to look out and pay attention to the potential ocular surface problems in patients with autoimmune diseases in the aspect of diagnosis. The clinical symptoms and signs and inflammation reactions of the dry eye caused by the autoimmune diseases became more general and more serious. The progression of autoimmune diseases was closely related to the progression of ocular surface diseases. Therefore, in the treatment, it is necessary to actively promote the functional recovery of lacrimal glands and the tear secretion as well as the repair of ocular surface epithelial cells, actively and effectively control the ocular surface inflammatory reactions, and actively and systematically treat the systemic autoimmune diseases. As for the severe patients of dry eye, a better therapeutic effect can be obtained only through the combination with the local immunosuppressive therapy. Because the patients with autoimmune diseases are in the state of unusual high immune response, the ocular surface inflammation reaction is serious, which increases the risk of producing the immune rejection after the corneal transplantation. For this reason, it must be very careful to conduct the corneal transplantation for the patients with autoimmune disease of serious dry eye, such as corneal dissolution and perforation. Therefore, it is a better choice to adopt the conjunctival flap covering surgery plus tarsorrhapy for patients of this kind. The next step of our study will elevate inflammatory mediators of ocular surface and tear film between IRDE and SDE.

## Figures and Tables

**Table 1:** Comparison of general data of two groups.

General data	SDE group	IRDE group			
		SS	SLE	RA	Total
Patients (person)	224	70	72	65	207
Male (person)	86^a^	2	3	17	22^b^
Female (person)	138^a^	68	69	48	185^b^
Average age (year)	53.6^c^	48.7	37.2	47.0	42.8^c^

^a^ *x* ^2^ = 5.71, *P* < 0.05; ^b^*x*^2^ = 31.63, *P* < 0.005; ^c^*t* = 2.965, *P* < 0.005.

**Table 2:** Comparison of signs.

Signs	SDE group	IRDE group			
		SS	SLE	RA	Total
TBUT (sec)	5.25 ± 0.67^a^	3.75 ± 0.38	4.25 ± 0.51	4.75 ± 0.35	4.25 ± 0.68^a^
Schirmer I test (mm/5 min)	6.14 ± 0.32^b^	3.25 ± 0.88	4.3 ± 0.75	4.73 ± 0.81	4.11 ± 0.76^b^
Corneal fluorescein staining scoring	5.25 ± 0.24^c^	10.25 ± 0.54	9.75 ± 0.89	8.60 ± 0.67	9.53 ± 0.82^c^

^a^ *t* = 1.282, *P* > 0.05; ^b^*t* = 2.453, *P* < 0.05; ^c^*t* = 3.636, *P* < 0.05.

**Table 3:** Comparison of ocular surface inflammatory reaction.

Inflammatory reaction signs	SDE group	IRDE group			
		SS	SLE	RA	Total
Conjunctival congestion ratio	32.2%^a^	69.3%	78.5%	63.1%	70.3%^a^
Conjunctival papillary hyperplasia ratio	27.5%^b^	52.4%	61.8%	50.8%	55.0%^b^
Conjunctival follicular hyperplasia ratio	16.3%^c^	36.8%	40.4%	39.2%	38.8%^c^
Secretion ratio	12.6%^d^	10.1%	13.6%	10.6%	11.4%^d^

^a^ *x* ^2^ = 15.33, *P* < 0.05; ^b^*x*^2^ = 11.09, *P* < 0.05; ^c^*x*^2^ = 7.82, *P* < 0.05; ^d^*x*^2^ = 0.32, *P*  >  0.05.

**Table 4:** Comparison of dry eye classification.

Classification	SDE group	IRDE group			
		SS	SLE	RA	Total
Case	224	70	72	65	207
Minor	87 (38.8%)	10 (14.3%)	18 (25.0%)	27 (41.5%)	55 (26.6%)
Moderate	77 (34.4%)	21 (30.0%)	26 (36.1%)	25 (38.5%)	72 (34.8%)
Severe	49 (21.9%)^a^	28 (40.0%)^c, d^	24 (33.3%)^c^	10 (15.4%)^d^	62 (30.0%)^a^
Extremely severe	11 (4.9%)^b^	11 (15.7%)^e, f^	4 (5.6%)^e^	3 (4.6%)^f^	18 (8.7%)^b^

^a^ *x* ^2^ = 7.21, *P* < 0.05; ^b^*x*^2^ = 12.39, *P* < 0.05; ^c^*x*^2^ = 6.76, *P* < 0.05; ^d^*x*^2^ = 15.61, *P* < 0.05; ^e^*x*^2^ = 8.66, *P* < 0.05; ^f^*x*^2^ = 9.13, *P* < 0.05.
